# Hidrox^®^ and Chronic Cystitis: Biochemical Evaluation of Inflammation, Oxidative Stress, and Pain

**DOI:** 10.3390/antiox10071046

**Published:** 2021-06-29

**Authors:** Ramona D’Amico, Angela Trovato Salinaro, Marika Cordaro, Roberta Fusco, Daniela Impellizzeri, Livia Interdonato, Maria Scuto, Maria Laura Ontario, Roberto Crea, Rosalba Siracusa, Salvatore Cuzzocrea, Rosanna Di Paola, Vittorio Calabrese

**Affiliations:** 1 Department of Chemical, Biological, Pharmaceutical and Environmental Sciences, University of Messina, 98166 Messina, Italy; rdamico@unime.it (R.D.); rfusco@unime.it (R.F.); dimpellizzeri@unime.it (D.I.); livia.interdonato@yahoo.it (L.I.); dipaolar@unime.it (R.D.P.); 2 Department of Biomedical and Biotechnological Sciences, University of Catania, 95123 Catania, Italy; trovato@unict.it (A.T.S.); mary-amir@hotmail.it (M.S.); marialaura.ontario@ontariosrl.it (M.L.O.); calabres@unict.it (V.C.); 3 Department of Biomedical, Dental and Morphological and Functional Imaging, University of Messina, Via Consolare Valeria, 98125 Messina, Italy; cordarom@unime.it; 4 Oliphenol LLC., 26225 Eden Landing Road, Unit C, Hayward, CA 94545, USA; robertocrea48@gmail.com; 5 Department of Pharmacological and Physiological Science, Saint Louis University School of Medicine, Saint Louis, MO 63104, USA

**Keywords:** chronic disease, inflammation, oxidative stress, pain, natural compound

## Abstract

Interstitial cystitis/painful bladder syndrome (IC/PBS) is a chronic bladder condition characterized by frequent urination, inflammation, oxidative stress, and pain. The aim of the study was to evaluate the anti-inflammatory and antioxidant effects of an oral administration of Hidrox^®^ (10 mg/kg) in the bladder and spinal cord in a rodent model of IC/BPS. The chronic animal model of cystitis was induced by repeated intraperitoneal injections of cyclophosphamide (CYP) for five consecutive days. Treatment with Hidrox^®^ began on the third day of the CYP injection and continued until the 10th day. CYP administration caused macroscopic and histological bladder changes, inflammatory infiltrates, increased mast cell numbers, oxidative stress, decreased expression of the tight endothelial junction (e.g., zonula occludens-1 (ZO-1) and occludin), and bladder pain. Treatment with Hidrox^®^ was able to improve CYP-induced inflammation and oxidative stress via the nuclear factor erythroid 2-related factor 2 (Nrf2)/heme oxygenase 1 (HO-1) pathway. It was also able to reduce bladder pain which was aggravated by the activation of neuroinflammation in the central nervous system. In particular, Hidrox^®^ reduced the brain-derived neurotrophic factor (BDNF), as well as the activation of astrocytes and microglia, consequently reducing mechanical allodynia. These results indicate that nutritional consumption of Hidrox^®^ can be considered as a new therapeutic approach for human cystitis, increasing the conceivable potential of a significant improvement in the quality of life associated with a lowering of symptom intensity in patients with IC/BPS.

## 1. Introduction

Interstitial cystitis/painful bladder syndrome (IC/BPS) is a chronic inflammatory condition of the bladder characterized by bladder pain and frequent urination [1]. The symptom of chronic pain seriously affects the quality of life of patients. IC/BPS affects all age groups of both sexes, although a 14-fold higher prevalence in females was observed compared to males [2]. To date, the etiology and the pathophysiology of this disease still remains to be clarified. Several theories have been proposed, including urothelial defects of the bladder or dysfunction, activation of mast cells, and autoimmunity [3]. Furthermore, some studies have highlighted that IC/BPS can be associated with stress or anxiety disorders that cause a sympathetic effect leading to inflammatory processes in IC [4]. The proliferation of epithelial cells and gene expression are often abnormal in the bladder fabric of patients with IC/BPS, with modified levels of inflammatory proteins, proteins involved in oxidative and nitrosative stress, and proteins of tight junctions such as zonula occludens-1 (ZO-1) and occludin [5].

Cyclophosphamide (CYP) is a drug widely used in the treatment of tumors and in the treatment of autoimmune diseases such as rheumatoid arthritis and systemic lupus erythematosus [6]. In addition to the beneficial effects of CYP, there are also numerous adverse effects. CYP is an inactive prodrug that is metabolized in acrolein and phosphoramide. It is known that acroleine increases the production of reactive oxygen species (ROS) and nitric oxide, leading to the formation of peroxynitrite which in turn damages lipids, proteins, and DNA inside the cell [7]. There are several animal models of well-described cystitis. Among the most used in rodents is the intraperitoneal injection (i.p.) of CYP, which produces bladder edema and bleeding, leading to pain-related behavior and mechanical hypersensitivity [8,9].

Recently, it was highlighted that cytokines, ROS, and modulation of transcription factors are involved in the pathogenesis of cystitis [10]. Therefore, it is important for the care of cystitis that compounds are discovered able to act on oxidative stress and inflammation, which are at the base of IC/BPS.

An interesting relationship between IC/BPS and pain behaviors has been established. Several studies have suggested that neuroinflammation can lead to an aggravation of pathological pain [11]. In particular, it has been reported that the brain-derived neurotrophic factor (BDNF) has a crucial role in the development of neuroinflammation [12]. An increase in the release of BDNF contributes to central awareness and the development of chronic pain [13]. Therefore, molecules capable of reducing the progress of pathology and raising pain awareness are eligible treatments for illness.

Notable is the scientific interest in hormesis, a biphasic dose–response characterized by low-dose stimulation and high-dose inhibition. We speak of hormesis when overcompensation reactions occur as a result of direct and immediate interruptions of cellular homeostasis induced by subtoxic or subthreshold doses of various stressors [14,15]. Such hormesis-induced overcompensation often improves cellular resistance [16,17]. Nuclear factor erythroid 2-related factor 2 (Nrf2) encodes the antioxidant pathway of vitagens, which exist to counter various forms of stress and help preserve protein homeostasis and cellular redox balance in various disease states [18,19,20]. The idea that Nrf2 can act as a hormetic mediator is interesting for the development of drugs and therapies.

Many studies have reported the beneficial effects of natural food phytocomponents and a Mediterranean diet (MD) in oxidative and painful diseases [21]. In particular, with regard to the MD, the study of olive oil is interesting for its rich content of bioactive compounds such as hydroxytyrosol (HT), which has been widely highlighted to have antioxidant and anti-inflammatory power [22,23]. It is interesting to note that HT is capable of activating Nrf2 by preserving the balance of cellular redox and homeostasis [24]. In our recent studies, it was shown that Hidrox^®^ (HD), an aqueous compound extracted from olive pulp containing 40–50% HT, was able to prevent the neurodegenerative progression of Parkinson’s disease through the Nrf2/HO-1 pathway [25]. Therefore, the purpose of this study was to evaluate the effect of HD administration in IC/BPS and the associated pain.

## 2. Materials and Methods

### 2.1. Animals

Female adult CD1 mice (25–30 g, Envigo, Casatenovo, Italy) were housed in a controlled location with free access to standard rodent food and water. The University of Messina Review Board for animal care (OPBA) approved the study. All the animal experiments agree with the new Italian regulations (D.Lgs 2014/26), EU regulations (EU Directive 2010/63), and the ARRIVE guidelines.

### 2.2. Chronic Experimental Cystitis Induction

Chronic cystitis was induced in mice by repetitive intraperitoneal (i.p.) injections of an alkylating antineoplastic agent (CYP). CYP (100 mg/kg) or vehicle (saline) was administered every day for 5 days. All mice were sacrificed by cervical dislocation 1 week after the third CYP injection [26]. A midline ventral abdominal incision was made to collect the bladder and next a longitudinal incision was made along the midline of the back to collect the L6-S1 area of the spinal cord.

#### Experimental Groups

Mice were randomly distributed into one of the following groups:

1. CYP + saline: Mice received CYP injections (an animal of 25 g received 250 μL of CYP dissolved in saline, i.p.) every day for 5 days and saline orally daily for 1 week (an animal of 25 g received 250 μL of saline), starting from the third day of CYP injection (*N* = 20);
2. CYP + HD: Mice received CYP (an animal of 25 g received 250 μL of CYP dissolved in saline, i.p.) every day for 5 days and HD orally (10 mg/Kg, an animal of 25 g received 250 μL of HD dissolved in saline) daily for 1 week, starting from the third day of CYP injection (*N* = 20);
3. Sham: Vehicle solution (saline) was administrated i.p. every day for 5 days (an animal of 25 g received 250 μL of saline), as in the CYP protocol, and saline was administered orally daily for 1 week (an animal of 25 g received 250 μL of saline), starting from the third day of saline injection (*N* = 20);
4. Sham + HD: Vehicle solution (saline) was administrated i.p. every day for 5 days (an animal of 25 g received 250 μL of saline), as in the CYP protocol, and HD was administered orally daily for 1 week (an animal of 25 g received 250 μL of HD dissolved in saline), starting from the third day of saline injection (*N* = 20).

The dose of Hidrox^®^ was based on previous experiments [25,27,28].

### 2.3. Macroscopic Analysis of Bladder Damage

After completing the experiment, the animals were sacrificed by cervical dislocation. The bladders were weighed and analyzed macroscopically to observe the formation of bleeding and edema. The score was established as follows, according to the previous work of Gray et al.: 3—severe damage (fluid externally present in the bladder wall and evident bleeding), 2—moderate damage (fluid in the internal mucosa and little bleeding), 1—mild damage (little edema and no bleeding), and 0—no bladder effect [27].

### 2.4. Assessment of Mechanical Hypersensitivity in Chronic Cystitis Model

Mechanical hypersensitivity in mice was measured at different time points using an electronic von Frey hair esthesiometer (Ugo Basile, Comerio, Italy) consisting of a portable force transducer equipped with a plastic tip [28]. Briefly, mice were placed in plastic boxes under which there was a metal mesh floor, and they were allowed to acclimatize for 15 min before starting the test. Thereafter, the transducer tip was placed perpendicular to the pelvic region until a sharp retraction of the abdomen, immediate licking or scratching of the area of stimulation, and jumping were observed. The mechanical threshold (expressed in grams) corresponding to the pressure that stimulated a behavioral reaction (withdrawal of the abdomen) was recorded automatically by the electronic device. The stimulation was applied three times, and the mean value was calculated as the mechanical threshold for each mouse.

### 2.5. Histological Evaluation

For histopathological examination, the bladders were taken 7 days after the third injection of CYP. The tissues were fixed at room temperature (RT) in buffered formaldehyde solution (10% in PBS); histological sections (7 μm) were stained with hematoxylin and eosin (H&E) and evaluated using a Leica DM6 microscope (Leica Microsystems SpA, Milan, Italy) equipped with a motorized stage and associated with Leica LAS X Navigator software (Leica Microsystems SpA, Milan, Italy) [29]. The stained sections were scored by two investigators in a blind fashion, and the level of inflammation was evaluated on a scale of 0–5 as follows: 0 = no inflammation, 1 = mild inflammation, 2 = mild/moderate inflammation, 3 = moderate inflammation, 4 = moderate/severe inflammation, and 5 = severe inflammation. The degree of fibrosis was evaluated by Masson’s trichrome method according to the manufacturer’s protocol (Bio-Optica, Milan, Italy) [30,31]. The quantity of fibrosis was evaluated as the percentage fibrotic area (blue staining) and quantified using image analysis software (Image J 1.8.0). Mast cell analysis was performed by toluidine blue staining [32].

### 2.6. Western Blot Analysis

Bladders and spinal cords were homogenized and Western blots were performed as previously described [33,34]. Specific primary antibodies (anti-Nrf2 (1:500, Santa Cruz Biotechnology, Santa Cruz, CA, USA, sc-365949), anti-HO-1 (1:500, Santa Cruz Biotechnology, sc-136960), anti-IL-1β (1:500, Santa Cruz Biotechnology, sc-52012, anti-IL-6 (1:500, Santa Cruz Biotechnology, sc-57315), or TNF-α (1:500, Santa Cruz Biotechnology, sc-52746)) were mixed in 5% *w/v* nonfat dried milk solution and incubated at 4 °C overnight (O/N). Next, blots were incubated with peroxidase-conjugated bovine anti-mouse IgG or peroxidase-conjugated goat anti-rabbit IgG secondary antibody (Jackson Immuno Research) for 1 h at room temperature. To verify the equal amounts of protein, membranes were also incubated with an antibody against β-actin or laminin (1:5000; Santa Cruz Biotechnology, C4 sc-47778, E1 sc-376248). The signals were captured with BIORAD ChemiDocTM XRS + software thanks to the use of a reagent that emits chemiluminescence (Super Signal West Pico, Pierce chemiluminescent substrate) [35]. The relative expression of the bands was subsequently normalized to β-actin levels. Image analysis was performed using Image Quant TL software, v2003 [32].

### 2.7. Immunohistochemical Analysis

Immunohistochemical localization of anti-occludin (1:100, Santa Cruz Biotechnology, sc-133256) or anti-ZO-1 (1:100, Santa Cruz Biotechnology, sc-33725) was performed in the bladder, while localization of anti-BDNF (1:100, Abcam, Cambridge, MA, USA, ab108319), anti-GFAP (1:200, Santa Cruz Biotechnology, sc-33673), or anti-Iba-1 (1:200, Santa Cruz Biotechnology, sc-32725) was investigated in the spinal cord. The sections were incubated O/N with primary antibodies, and then all sections were washed with PBS and treated as previously reported [36]. Stained sections were observed using a Leica DM6 microscope following a typical procedure. The histogram profile was related to the positive pixel intensity value obtained by the computer program [37]. Immunohistochemical analyses were performed by experienced people who were unfamiliar with the treatment.

### 2.8. Preparation of the Bladder Homogenate for the Evaluation of Oxidative Stress

Bladder tissue was collected and transferred to very cold phosphate-buffered saline (pH 7.4). It was then cut into thin slices with a surgical scalpel, suspended in a cooled sucrose solution (0.25 M), and dried quickly on filter paper. Tissues were homogenized to release soluble proteins in a cooled tris hydrochloride buffer (10 mM, pH 7.4), and the homogenate was centrifuged at 7000 rpm for 20 min to obtain the supernatant, which was used for the estimation of oxidative stress parameters.

#### 2.8.1. Estimation of Lipid Peroxidation

Malondialdehyde (MDA) levels in the bladder samples were determined as an indicator of lipid peroxidation as previously described [38,39]. Briefly, 200 μL of homogenate was mixed with 200 μL of 8.1% sodium dodecyl sulfate, 1.5 mL of 30% acetic acid, and 1.5 mL of 0.8% of thiobarbituric acid. The mixture was heated for 60 min at 95 °C and subsequently rapidly cooled on ice. After cooling, 1.0 mL of distilled water and 5.0 mL of an n-butanol/pyridine solution (15:1 *v*/*v*) were added to each tube, before immediately centrifuging at 5000 rpm for 20 min. The levels of MDA were determined using a microplate reader at 532 nm and expressed as μg/mg of protein.

#### 2.8.2. Estimation of Superoxide Dismutase (SOD)

Bladder tissue determination of SOD activity was performed according to a previously described method [40]. Briefly, to 100 μL of tissue supernatant, 2.85 mL of phosphate buffer (0.1 M, pH 8.4) and 50 μL of pyrogallol (7.5 mM) were added. Enzyme levels were expressed as SOD activity in units per mg protein and were determined using a microplate reader at 420 nm.

#### 2.8.3. Estimation of Glutathione

The levels of GSH and GSSG in the bladder tissue were measured using GSH and GSSG Assay Kits (Beyotime, China) based on the method described by Tietze [41], according to the kit manufacturer’s instructions. GSH/GSSG ratio levels were expressed in μg/mg protein.

#### 2.8.4. Estimation of Catalase (CAT)

The bladder tissue determination of CAT activity was performed according to a previously described method [42]. Briefly, 50 μL of tissue supernatant was withdrawn, and 1.0 mL of phosphate buffer (50 mM, pH 7) and 100 μBL of hydrogen peroxide (30 mM) were added to it. Enzyme levels were expressed as CAT activity in units per mg protein and were determined using a microplate reader at 240 nm.

### 2.9. Statistical Analysis

All values in the figures and text are expressed as the mean ± standard deviation (SD) of *N* observations. For the in vivo studies, *N* represents the number of animals studied. In experiments involving histology, the figures shown are representative of at least three experiments performed on different days on tissue sections collected from all animals in each group. The results were analyzed by one-way ANOVA followed by a Bonferroni post hoc test for multiple comparisons. A *p*-value < 0.05 was considered significant.

## 3. Results

### 3.1. Effects of HD on Bladder Damage and Fibrosis after Repeated CYP Injections

**Figure 1:**
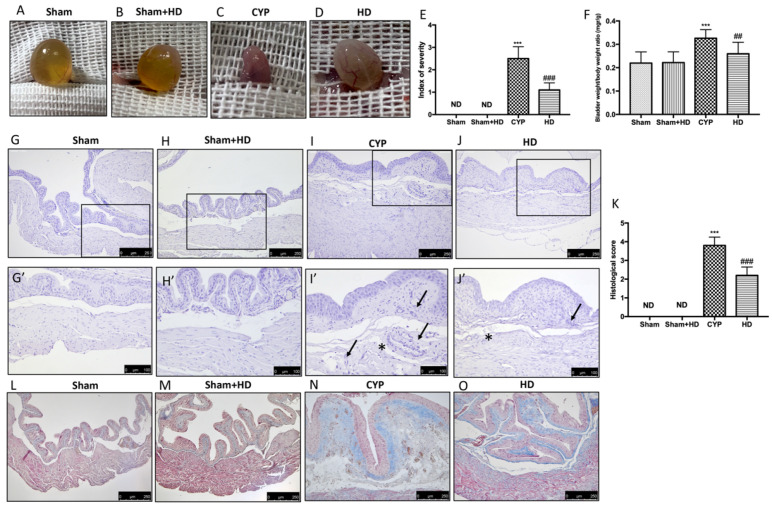
Effect of Hidrox^®^ (HD) on macroscopic and microscopic damage of bladder. Macroscopic damage in Sham mice (**A**,**E**), Sham + HD mice (**B**,**E**), mice with injection of CYP (**C**,**E**) and mice treated with HD (**D**,**E**). (**F**) Bladder weight/body weight ratio. Histological evaluation (**H**,**E** staining): Sham mice (**G**,**G’**,**K**), Sham + HD group (**H**,**H’**,**K**), CYP group (**I**,**I’**,**K**), and HD group (**J**,**J’**,**K**). Symbols used in images I’ and J’ show edema (asterisk) and cell infiltration (black arrow). Fibrosis evaluation (Masson’s trichrome stain): Sham mice (**L**), Sham + HD (**M**), CYP mice (**N**), and HD mice (**O**). Values are means ± SD of five animals for each group; *** *p* < 0.001 vs. Sham; ^##^ *p* < 0.01 vs. CYP; ^###^ *p* < 0.001 vs. CYP; ND: not detectable.

Edema and bleeding in the bladders were observed in mice administered CYP (Figure 1C,E), compared with the Sham and Sham + HD groups (Figure 1A,B,E). The oral administration of HD reduced this bladder damage (Figure 1D,E). In the Supplementary Materials, we show the data of the macroscopic analysis carried out on all the animals (Table S1). Furthermore, repeated injections of CYP increased bladder weight compared to control animals. HD reported a ratio of bladder weight to body weight closer to the Sham group (Figure 1F). Histopathological examination of the bladder showed alterations after CYP injection. Severe signs of cystitis including inflammatory cell infiltrate and submucosal edema were evident 7 days after the third injection of CYP (Figure 1I,I’; see histological score K). In control mice and in Sham + HD mice, an intact urothelium and normal muscularis were evident with no signs of edema (Figure 1G,G’ and H,H’; see histological score K). HD treatment significantly reduced histological damage (Figure 1J,J’; see histological score K). Masson’s trichrome stain revealed a noticeable increase in fibrosis in the inflamed bladders (Figure 1N) compared to the Sham and Sham + HD groups (Figure 1L,M). The degree of fibrosis (blue-colored fibrotic area) was reduced by HD treatment (Figure 1O).

### 3.2. Effects of HD on Oxidative Stress after Repeated CYP Injections

**Figure 2:**
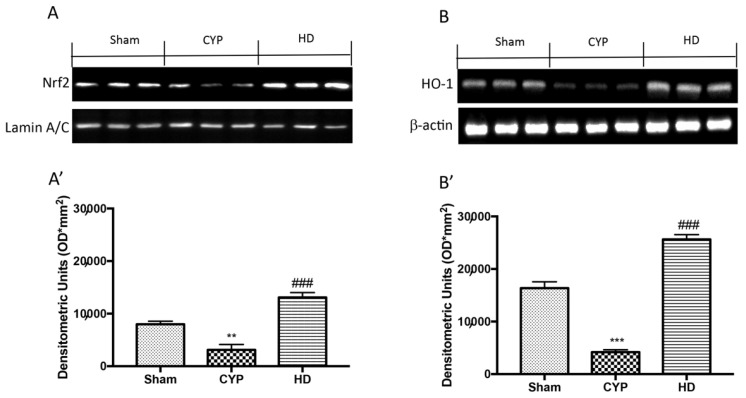
Effect of HD on Nrf2 and HO-1 expression in bladder after repeated CYP injections. Western blot analysis showed that Nrf2 expression was significantly decreased in the CYP group, while treatment with HD increased the expression of Nrf2 (**A**,**A’**). HO-1 expression was decreased after repeated CYP injections; treatment with HD increased levels of this protein (**B**,**B’**). Values are means ± SD of five animals for each group; ** *p* < 0.01 vs. Sham; *** *p* < 0.001 vs. Sham; ^###^ *p* < 0.001 vs. CYP.

To understand the effect of HD on cellular stress response, we evaluated the action of this compound on Nrf2 and HO-1. The results show a reduction in the expression of Nrf2 and HO-1 in the bladders of mice treated with CYP, compared to control mice that instead showed basal levels of these proteins. Instead, the HD treatment induced an increase in the expression of both Nrf2 and HO-1 (Figure 2A,A’ and 2B,B’, respectively).

**Figure 3:**
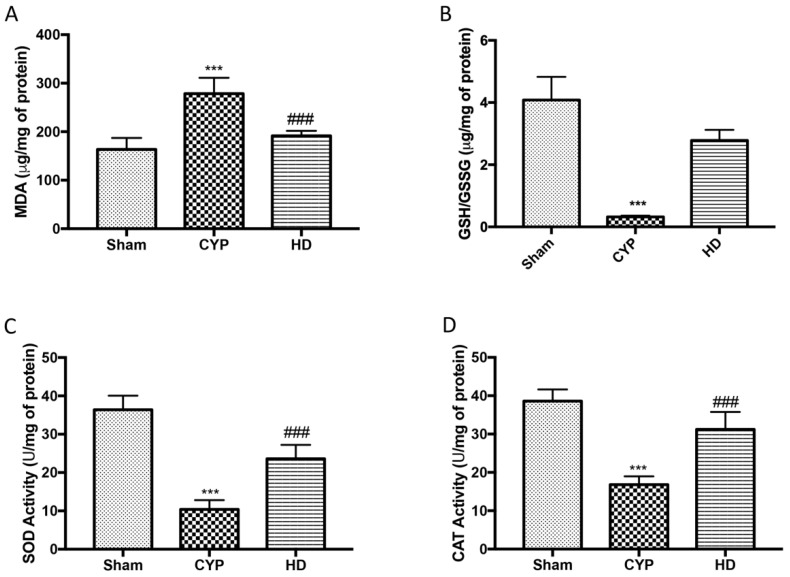
Effect of HD on oxidative stress in CYP-induced chronic cystitis: (**A**) Malondialdehyde content (MDA); (**B**) reduced glutathione/oxidized glutathione ratio (GSH/GSSG); (**C**) superoxide dismutase (SOD); (**D**) catalase. Data are expressed as the mean ± SD of five animals for each group; *** *p* < 0.001 vs. Sham; ^###^ *p* < 0.001 vs. CYP.

A state of marked oxidative stress in the CYP group was also observed by measuring the levels of MDA as a marker of lipid peroxidation, as well as the levels of reduced glutathione/oxidized glutathione ratio (GSH/GSSG) and the activity of SOD and CAT as markers of antioxidant defense systems. In particular, a significant increase in MDA levels and a consistent reduction in GSH levels and in the activity of SOD and CAT in the bladders taken from mice treated with CYP were highlighted. Treatment with HD alleviated the effect both on MDA and GSH/GSSG, and on SOD and CAT activity (Figure 3A–D).

### 3.3. Effects of HD on Bladder Inflammation and on Tight Junction (TJ) after Repeated CYP Injections

**Figure 4:**
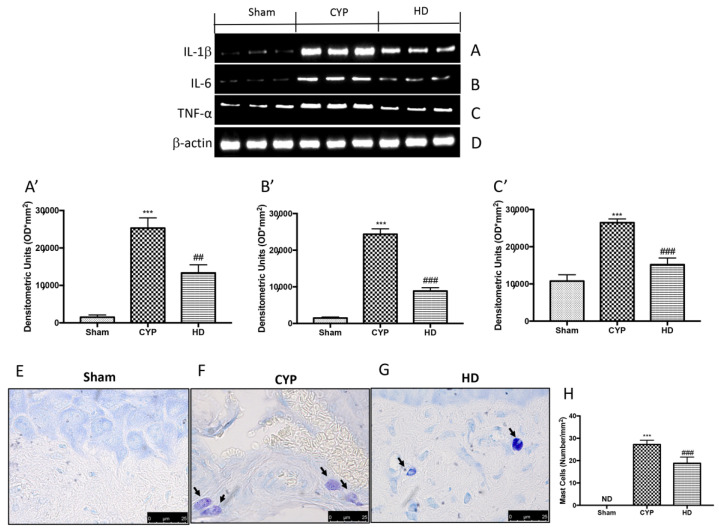
Effect of HD on the release of proinflammatory cytokines and mast cell (MC) activation in the bladders of mice after repeated CYP injections. Western blot analysis showed that IL-1β expression was significantly increased in the CYP group, while treatment with HD decreased the expression of IL-1β (**A**,**A’**). IL-6 expression was increased after repeated CYP injections; treatment with HD decreased levels of this cytokine (**B**,**B’**). TNF-α expression was significantly increased in the CYP group, while treatment with HD decreased the expression of TNF-α (**C**,**C’**). Mast cell infiltration was evaluated by toluidine blue staining in the chronic cystitis model. Mast cells were characterized by dark-lilac-blue granules. Protein lysates were also incubated with a β-actin antibody (**D**) in order to verify that all samples had been loaded in equal quantities. (**E**) Sham group. (**F**) CYP group. (**G**) HD group. (**H**) Mast cell numbers per unit area of tissue (mast cell density). Values are means ± SD of five animals for each group; *** *p* < 0.001 vs. Sham; ^##^ *p* < 0.01 vs. CYP; ^###^ *p* < 0.001 vs. CYP.

To investigate whether HD was capable of acting on the inflammatory processes that are activated during chronic cystitis, we evaluated the release of inflammatory cytokines by Western blot analysis and the activation of mast cells (MCs) using toluidine blue staining. We observed a marked increase in the expression of cytokines IL-1β, IL-6, and TNF-α in the bladder tissue in the group treated with CYP compared to the Sham group. The HD-treated group showed decreased levels of IL-1β, IL-6, and TNF-α, demonstrating an inhibitory effect on the release of proinflammatory cytokines (Figure 4A,A’, 4B,B’, and 4C,C’, respectively). An increase in the number and activation of MCs was found in the bladders of mice treated with CYP (Figure 4F,H), compared to control mice (Figure 4E,H). HD significantly reduced the number of MCs (Figure 4G,H).

**Figure 5:**
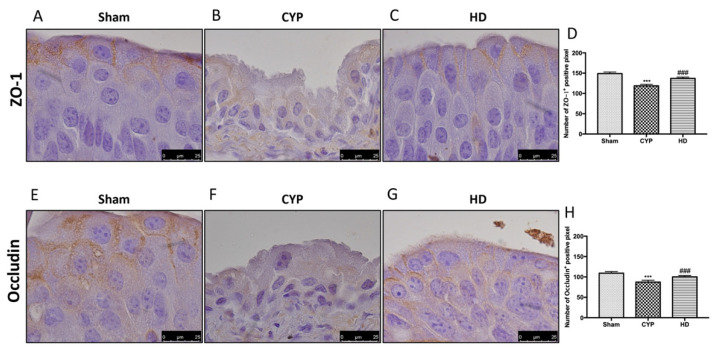
Effect of HD treatment on ZO-1 and occludin expression after chronic cystitis. Immunohistochemical analysis showed a significant loss of ZO-1-positive cells (**B**,**D**) compared to Sham mice (**A**,**D**). Animals treated with HD revealed an increase in the expression of ZO-1 (**C**,**D**). Immunohistochemical analysis for occludin displayed a significant loss of occludin-positive cells (**F**,**H**) compared to Sham mice (**E**,**H**). Mice treated with HD revealed an increase in the expression of occludin (**G**,**H**). Data are expressed as the number of ZO-1-positive pixel and occludin-positive pixels and are the means ± SD of five animals for each group. *** *p* < 0.001 vs. Sham; ^###^ *p* < 0.001 vs. CYP.

There is a substantial body of evidence showing changes in the expression of TJ-associated proteins in biopsies from patients with IC/BPS [43]. A decrease in the expression of ZO-1 and occludin was observed in the bladders of mice injected with CYP (Figure 5B,F and 5D,H, respectively), compared to the control groups (Figure 5A,E and 5D,H, respectively). Treatment with HD significantly reduced this TJ alteration (Figure 5C,G and 5D,H, respectively).

### 3.4. Effects of HD on Neuroinflammation after Repeated CYP Injections

**Figure 6:**
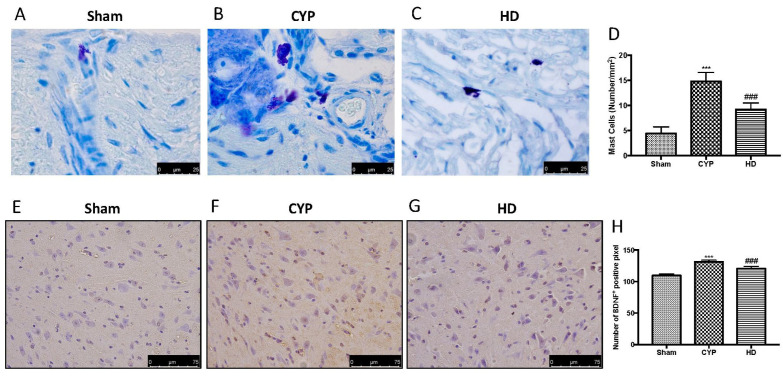
Effect of HD on MC activation and brain-derived neurotrophic factor (BDNF) expression in the spinal cord after repeated CYP injections**.** Mast cell infiltration was evaluated by toluidine blue staining in the chronic cystitis model. Mast cells were characterized by their dark-lilac-blue granules. (**A**) Sham group. (**B**) CYP group. (**C**) HD group. (**D**) Mast cell numbers per unit area of tissue (mast cell density). Immunohistochemical analysis showed a significant increase in BDNF-positive cells (**F**,**H**) compared to Sham mice (**E**,**H**). Animals treated with HD revealed a decrease in the expression of BDNF (**G**,**H**). Values are means ± SD of five animals for each group; *** *p* < 0.001 vs. Sham; ### *p* < 0.001 vs. CYP.

To demonstrate that CYP-induced alterations in the bladder also cause changes in the central nervous system, we evaluated the activation of MCs, the expression of BDNF, GFAP, and Iba-1, and the release of proinflammatory cytokines such as IL-1β and TNF-α in the L6-S1 area of the spinal cord. MC infiltration and degranulation were assessed by toluidine blue staining. In the spinal cord, a significant upregulation of the number of MCs, which play a key role in the inflammatory process, was observed in the mice treated with CYP (Figure 6B,D), compared to the control group (Figure 6A,D). Treatment with HD reduced the number of MCs in the L6-S1 area of the spinal cord (Figure 6C,D). Immunohistochemical analysis of BDNF showed an upregulation of this protein in the group treated with CYP (Figure 6F,H), compared to the basal levels of the Sham group (Figure 6E,H). Treatment with HD significantly reduced BDNF levels in the spinal cord (Figure 6G,H).

**Figure 7:**
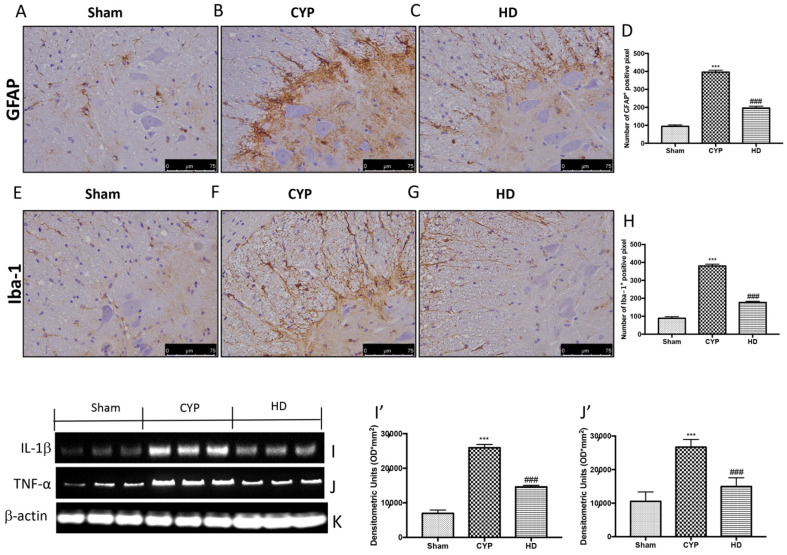
Effect of HD on neuroinflammation and on the release of proinflammatory cytokines in the spinal cord after repeated CYP injections. Immunohistochemical analysis showed a significant increase in glial fibrillary acidic protein (GFAP)-positive cells (**B**,**D**) and Iba-1-positive cells (**F**,**H**), compared to Sham mice (**A**,**D** for GFAP and **E**,**H** for Iba-1). Mice treated with HD revealed a decrease in the expression of GFAP (**C**,**D**) and Iba-1 (**G**,**H**). Data are expressed as the number of GFAP- and Iba-1-positive pixels and are the means ± SEM of five animals for each group. Western blot analysis showed that IL-1β expression was significantly increased in the CYP group, while treatment with HD decreased the expression of IL-1β (**I**,**I’**). TNF-α expression was significantly increased in the CYP group, while treatment with HD decreased the expression of TNF-α (**J**,**J’**). Protein lysates were also incubated with a β-actin antibody (**K**) in order to verify that all samples had been loaded in equal quantities. Values are means ± SD of five animals for each group; *** *p* < 0.001 vs. Sham; ^###^ *p* < 0.001 vs. CYP.

In addition, significant positive staining of GFAP and Iba-1 was observed in the spinal cord of the CYP group mice (Figure 7B,F and 7D,H, respectively), compared to the control mice (Figure 7A,E and 7D,H, respectively). Treatment with HD decreased the activation of both astrocytes and microglia (Figure 7C,G and 7D,H, respectively). Lastly, through Western blot analysis, we demonstrated that repeated CYP injections induced the release of IL-1β and TNF-α, whereas, in the Sham group, the levels of these inflammatory cytokines were low. Treatment with HD significantly prevented the release of IL-1β and TNF-α (Figure 7I,J and 7I’,J’, respectively).

### 3.5. Effects of HD on Mechanical Allodynia in Chronic Cystitis

**Figure 8:**
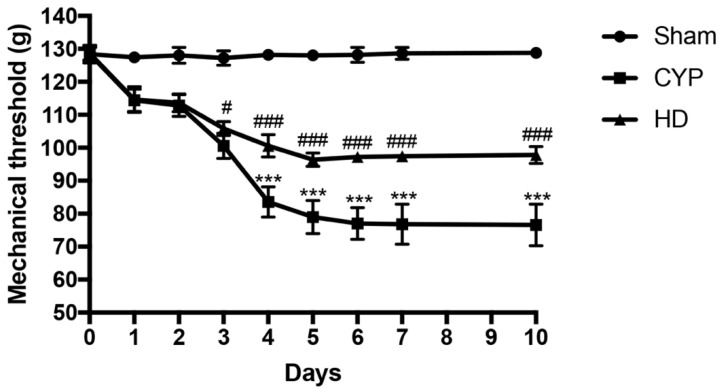
Effect of HD on mechanical allodynia in CYP-injected mice. Mechanical allodynia was evaluated by von Frey test. Values are means ± SD of five animals for each group; *** *p* < 0.001 vs. Sham; ^#^ *p* < 0.05 vs. CYP; ^###^ *p* < 0.001 vs. CYP.

We investigated the effects of HD on pain associated with chronic cystitis using the von Frey test. Repeated injections of CYP gradually reduced the withdrawal threshold in response to abdominal stimulation, reaching its lowest value around day 7 and persisting until day 10. Treatment with HD resulted in a significant improvement in pain-related responses (Figure 8).

## 4. Discussion

Hydroxytyrosol is a polyphenyl that commonly induces hormetic dose responses in a wide range of biological models. Hydrox^®^, which contains 40–50% hydroxytyrosol, has been shown in our recent study to have hormetic properties and to induce the activation of the phase II enzyme responsible for antioxidant responses through the activation of the Nrf2/HO-1 pathway [25,44]. This pathway represents one of the antioxidant systems most involved in the maintenance of the intracellular redox state and, therefore, is widely studied for the prevention of numerous diseases caused by oxidative stress [45,46]. It is well-documented that CYP-induced cystitis induces an increase in oxidative stress and inflammation in the bladder [47,48]. Our study showed that treatment with HD through the overexpression of Nrf2 and HO-1 restored the levels of GSH, SOD, CAT, and reduced lipid myeloperoxidation. All these results indicate that HD is capable of reducing the oxidative stress induced by CYP treatment. Connected to oxidative stress is the activation of mast cells and the inflammatory response with the release of pro-inflammatory cytokines and chemokines in the bladder [49,50,51,52,53]. HD, in addition to having high antioxidant power, is able to act on inflammatory processes [25]. In the present study, we demonstrated the ability of HD to significantly reduce the number of activated MCs and the consequent release of IL-1β, IL-6 and TNF-α. The effect of HD on oxidative stress and bladder inflammation led to a significant reduction in histopathological damage and loss of tight junctions whose function is important as they prevent pathogens from entering the bladder which would lead to urinary disease [54]. Several reports have indicated that inflammation and in particular the increased number of MCs in the bladder increase afferent sensitivity and nociception, contributing to symptoms of urinary frequency, urgency and pelvic pain in patients with IC/BPS [55]. This is due to the interaction between the immune system and the neural system, known as the neuroimmune interface [56,57,58,59]. Our study demonstrated the presence of activated MCs in the L6-S1 region of the spinal cord of mice treated with CYP. Migration of MCs induced the release of proinflammatory cytokines such as IL-1β and TNF-α also in the spinal cord where they are responsible for pelvic pain [60,61,62]. Another factor that together with MCs seems to play a crucial role in increasing urinary frequency and inducing the neuroinflammation responsible for pelvic pain perceived in patients with IC/BPS is the neurotrophic factor BDNF [12,59,62]. Our results on molecular changes in the spinal cord demonstrated that HD is able to reduce the activation of MCs, as well as the levels of BDNF, GFAP, Iba-1, IL-1β, and TNF-α also in the spinal cord and consequently reduce CYP-induced mechanical allodynia.

## 5. Conclusions

HD, by modulating oxidative stress, reduces bladder inflammation by suppressing mast cell proliferation and inhibiting the release of proinflammatory mediators. This prevents mast cell migration into the spinal cord and central hypersensitivity, leading to neuroinflammation and increased mechanical allodynia. Therefore, Hidrox^®^ could be used as a dietary supplement to counter IC/BPS and improve the quality of life of patients.

## Data Availability

The data presented in this study are available upon request of the corresponding author. The data is not publicly available due to the internal rules of the research group.

## Supplementary Materials

The following are available online at https://www.mdpi.com/2076-3921/10/7/1046/s1. Table S1: Raw data of macroscopic analysis.
