# Correction: D’Amico et al. Hidrox^®^ and Chronic Cystitis: Biochemical Evaluation of Inflammation, Oxidative Stress, and Pain. *Antioxidants* 2021, *10*, 1046

**DOI:** 10.3390/antiox14091106

**Published:** 2025-09-11

**Authors:** Ramona D’Amico, Angela Trovato Salinaro, Marika Cordaro, Roberta Fusco, Daniela Impellizzeri, Livia Interdonato, Maria Scuto, Maria Laura Ontario, Roberto Crea, Rosalba Siracusa, Salvatore Cuzzocrea, Rosanna Di Paola, Vittorio Calabrese

**Affiliations:** 1 Department of Chemical, Biological, Pharmaceutical and Environmental Sciences, University of Messina, 98166 Messina, Italy; rdamico@unime.it (R.D.); rfusco@unime.it (R.F.); dimpellizzeri@unime.it (D.I.); livia.interdonato@yahoo.it (L.I.); dipaolar@unime.it (R.D.P.); 2 Department of Biomedical and Biotechnological Sciences, University of Catania, 95123 Catania, Italy; trovato@unict.it (A.T.S.); mary-amir@hotmail.it (M.S.); marialaura.ontario@ontariosrl.it (M.L.O.); calabres@unict.it (V.C.); 3 Department of Biomedical, Dental and Morphological and Functional Imaging, University of Messina, Via Consolare Valeria, 98125 Messina, Italy; cordarom@unime.it; 4 Oliphenol LLC., 26225 Eden Landing Road, Unit C, Hayward, CA 94545, USA; robertocrea48@gmail.com; 5 Department of Pharmacological and Physiological Science, Saint Louis University School of Medicine, Saint Louis, MO 63104, USA

In the original publication [1], there was an error in Figure 6C. The authors unintentionally included an incorrect figure. Specifically, in Figure 6, for the group HD, they inadvertently attached the wrong picture.

The authors checked all the data in their laboratory, found the original photos, and prepared a revised figure using an appropriate representative image from their database, belonging to the experimental group in question.

The authors apologize for any inconvenience caused by this oversight. The new Figure 6 appears below. The authors state that the scientific conclusions are unaffected. This correction was approved by the Academic Editor. The original publication has also been updated.

**Figure 6:**
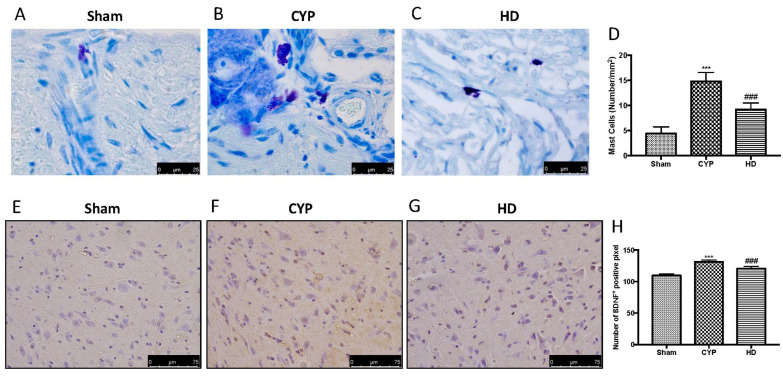
Effect of HD on MC activation and brain-derived neurotrophic factor (BDNF) expression in the spinal cord after repeated CYP injections. Mast cell infiltration was evaluated by toluidine blue staining in the chronic cystitis model. Mast cells were characterized by their dark-lilac-blue granules. (**A**) Sham group. (**B**) CYP group. (**C**) HD group. (**D**) Mast cell numbers per unit area of tissue (mast cell density). Immunohistochemical analysis showed a significant increase in BDNF-positive cells (**F**,**H**) compared to Sham mice (**E**,**H**). Animals treated with HD revealed a decrease in the expression of BDNF (**G**,**H**). Values are means ± SD of five animals for each group; *** *p* < 0.001 vs. Sham; ### *p* < 0.001 vs. CYP.
